# The Proteins behind the Persistence of Memory

**DOI:** 10.1371/journal.pbio.1001787

**Published:** 2014-02-11

**Authors:** Richard Robinson

**Affiliations:** Freelance Science Writer, Sherborn, Massachusetts, United States of America

At its most fundamental level, a memory is an increase in synaptic strength that persists over time. That persistence requires synthesis of a specific set of synaptic proteins, a process regulated by so-called Cytoplasmic Polyadenylation Element Binding (CPEB) proteins. The remarkable feature of these proteins is their prion-like nature. Their conversion from monomers to oligomers, via stacking of their prion domains, is essential to the long-term maintenance of synaptic memory.

**Figure:**
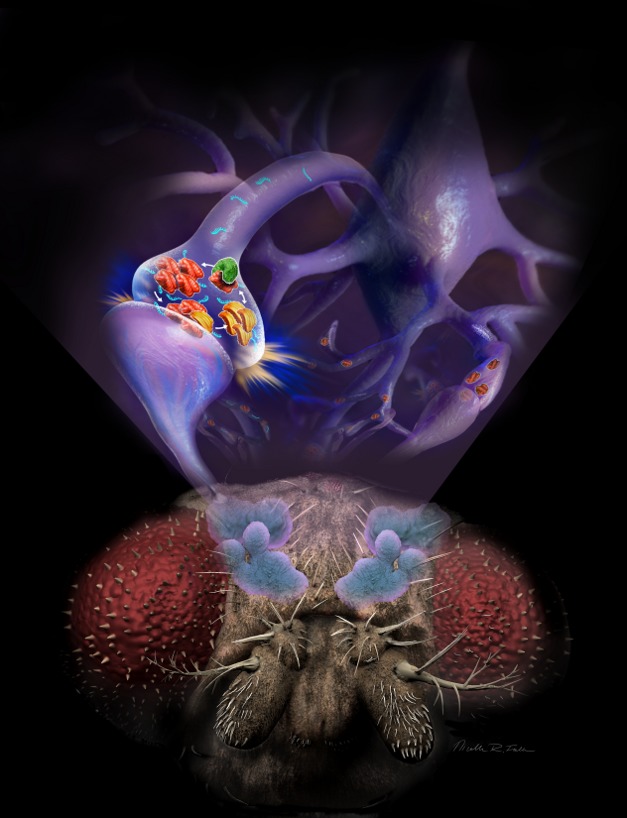
Tob (green) binds and increases the amount of monomeric Orb2A (red), which facilitates conformational change and oligomerization of Orb2 (yellow). *Image Credit: Nicolle Rager Fuller, Sayo-Art*.

But in order to remember the right things, and not the wrong ones, the location and timing of oligomerization must be tightly regulated. In the current issue of *PLOS Biology*, Erica White-Grindley, Kausik Si, and colleagues use the fruit fly to reveal several essential players in that regulatory process and provide a compelling outline for understanding how, and when, memories form.

The CPEB protein at work in the fruit fly is Orb2, which occurs in two isoforms: Orb2A and Orb2B. Orb2A, by far the rarer of the two, has a prion-like domain, so the authors began by searching for Orb2A binding partners. They found quite a few, but one in particular, called Tob, intrigued them, in part because it also appeared to regulate Orb2A levels, which suggested Tob might stabilize the usually labile Orb2A. Indeed, increasing Tob almost doubled the half-life of Orb2A and reducing it depleted Orb2A (neither action had an effect on Orb2B). Overexpressing Tob also increased the concentration of Orb2 oligomers. Furthermore, activation of fly neurons enhanced the association of Tob and Orb2A, suggesting their linkage plays a role in response to synaptic activity.

One well-characterized long-term memory in fruit flies is courtship behavior suppression, induced in males by repeated exposure to an unreceptive female. Males treated with RNAi against Tob persisted in courting such females, indicating their inability to form oligomer-dependent long-term memories. Tob, then, was clearly playing a central role in memory stabilization.

But Tob is always present in the neuron, so how can it provide spatial and temporal control on Orb2 oligomerization? The authors discovered that the Orb2A-Tob complex also contained the phosphatase PP2A, whose job is to remove phosphates. When they blocked dephosphorylation, they reduced Orb2A-Tob association. This destabilized Tob but, intriguingly, stabilized Orb2A, suggesting that part of PP2A's role is to promote degradation of unbound Orb2A.

If loss of phosphate destabilizes Orb2A, and if Tob's role is to stabilize Orb2A and enhance its oligomerization, one way Tob might do so would be to promote addition of phosphates to Orb2A. The authors found that LimK, a kinase acting on Tob, also phosphorylated Orb2A when Orb2A was linked to Tob. That phosphorylation was also critical to the oligomerization process; when LimK was present but inactivated, there was no increase in oligomerization in response to neuronal stimulation.

The model the authors propose based on these results posits that in the unstimulated synapse, PPA destabilizes newly synthesized Orb2A and prevents oligomerization. Among many other changes, synaptic stimulation increases Orb2A synthesis, through unknown mechanisms. Excess Orb2A binds to Tob, which temporarily stabilizes it while recruiting LimK. This triggers oligomerization, including of the more abundant Orb2B. The presence of Orb2 oligomers maintains the translational changes underlying the change in synaptic strength. Those translational changes, originally brought about by stimulation, are now preserved as a new memory.

Much remains unknown about this dynamic system, including whether and how Tob and LimK may be involved in regulating the Orb2-dependent translation that follows oligomerization. But with the identification of this important regulatory system, it is likely that a more detailed understanding of post-stimulation memory maintenance will emerge relatively quickly.

**White-Grindley E, Li L, Khan RM, Ren F, Saraf A, et al. (2014) Contribution of Orb2A Stability in Regulated Amyloid-Like Oligomerization of** ***Drosophila*** **Orb2.** doi:10.1371/journal.pbio.1001786

